# Chiral Magnonics: Reprogrammable Nanoscale Spin Wave Networks Based on Chiral Domain Walls

**DOI:** 10.1016/j.isci.2020.101153

**Published:** 2020-05-12

**Authors:** Jilei Chen, Junfeng Hu, Haiming Yu

**Affiliations:** 1 Fert Beijing Institute, BDBC, School of Microelectronics, Beihang University, Beijing, China

**Keywords:** Physics, Magnetism, Computational Materials Science

## Abstract

Spin waves offer promising perspectives as information carriers for future computational architectures beyond conventional complementary metal-oxide-semiconductor (CMOS) technology, owing to their benefits for device minimizations and low-ohmic losses. Although plenty of magnonic devices have been proposed previously, scalable nanoscale networks based on spin waves are still missing. Here, we demonstrate a reprogrammable two-dimensional spin wave network by combining the chiral exchange spin waves and chiral domain walls. The spin-wave network can be extended to two dimensions and offers unprecedented control of exchange spin waves. Each cell in the network can excite, transmit, and detect spin waves independently in the chiral domain wall, and spin-wave logics are also demonstrated. Our results open up perspectives for integrating spin waves into future logic and computing circuits and networks.

## Highlights

• Two-dimensional reprogrammable nanoscale spin-wave networks are demonstrated
• The networks allow the control of chiral spin waves in chiral domain walls
• Logic gates are realized in nanoscale spin-wave networks

## Graphical Abstract

**Figure:**
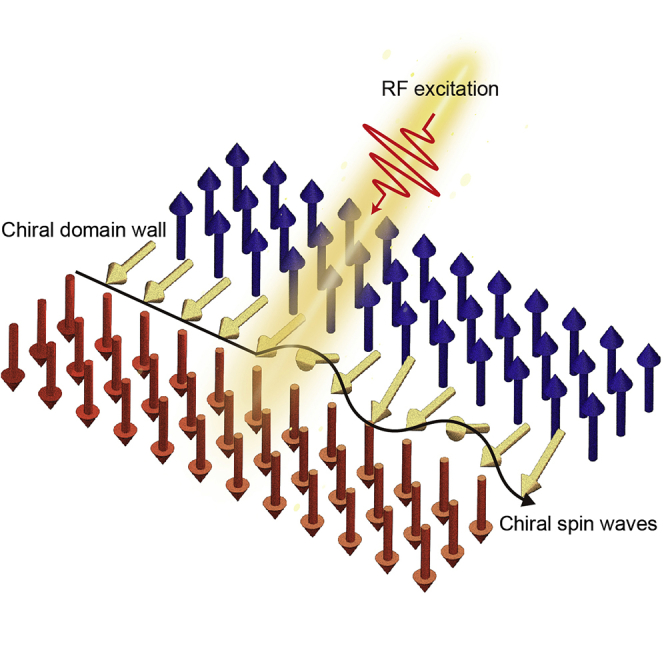

## Introduction

As elementary spin excitations in magnetic materials, spin waves are promising candidates as information carriers for the future computational architectures (Chumak et al., 2015, Kruglyak et al., 2010, Demidov et al., 2017). Spin waves can transmit information free of charge transport and therefore enable the realization of low-power-consumption devices in the absence of Joule heating (Wang et al., 2019, Han et al., 2019, Kajiwara et al., 2010, Cornelissen et al., 2015, Liu et al., 2018, Qin et al., 2018, Khitun and Wang, 2005, Csaba et al., 2017). Compared with dipolar spin waves, exchange spin waves possess much shorter wavelengths (*λ* < 100 nm), higher group velocities, and higher resonance frequencies (Kalinikos and Slavin, 1986). Exchange spin waves with wavelengths around 10 nm exhibit a high group velocity of ~13.1 km/s, ten times faster than the dipolar spin waves in a thin-film magnetic insulator (Liu et al., 2018). The demand of high signal processing speed in modern nano-electronic devices makes exchange spin waves important for future applications (Dieterle et al., 2019, Che et al., 2020). Recently, experimental observations of exchange spin waves via magnetic nano-arrays have opened a horizon for applying exchange spin waves in magnonic devices and circuits (Yu et al., 2016). In addition, chiral excitation of exchange spin waves allows unidirectional spin-wave propagation (Chen et al., 2019b), which is key to the realization of magnonic logic devices and circuits. Although plenty of magnonic devices have been proposed previously, such as magnonic transistors (Chumak et al., 2014), diodes (Lan et al., 2015), and directional couplers (Wang et al., 2018), scalable nanoscale magnonic networks are still missing.

The Dzyaloshinskii-Moriya interaction (DMI)-induced breaking of chiral symmetry has triggered many significant breakthroughs in spintronics, including magnetic skyrmions (Mühlbauer et al., 2009, Nagaosa and Tokura, 2013, Fert et al., 2017) and chiral domain walls (Ryu et al., 2013, Emori et al., 2013, Siddiqui et al., 2018). Recently, spin waves have been found to interact with magnetic domain walls (Wagner et al., 2016), antiferromagnetically coupled domains (Liu et al., 2019), and skyrmion strings (Seki et al., 2020), which open up an era for the spin textures-based magnonic applications. The interactions between spin waves and magnetic domain walls are widely investigated in the field of spintronics (Han et al., 2019, Pirro et al., 2015, Woo et al., 2017, Chang et al., 2018, Hämäläinen et al., 2018, Körber et al., 2017, Zhang et al., 2018, Yan et al., 2011, Buijnsters et al., 2016, Sluka et al., 2019, Jamali et al., 2010). In magnetic systems with the perpendicular magnetic anisotropy (PMA), Néel-type domain walls with left- or right-handed chiralities can be formed in the presence of the interfacial DMI (i-DMI) (Ryu et al., 2013, Emori et al., 2013). When spin waves propagate inside a chiral domain wall, spins in the wall are perpendicular to the wavevector, where a quasi-Damon-Eshbach (DE) mode spin waves can transport efficiently (Garcia-Sanchez et al., 2015). Meanwhile, owing to the particular spin textures, spin waves are able to propagate inside the domain walls without applied external fields, which benefits the high-energy-efficient magnonic applications. More recently, the i-DMI has been discovered in the magnetic insulator thin films with PMA, which possesses low damping compared with metallic films (Soumah et al., 2018, Chen et al., 2019a), and Néel-type domain walls (Avci et al., 2019, Vélez et al., 2019) as well as magnetic skyrmions (Shao et al., 2019, Ding et al., 2019) are also observed. The i-DMI is found to be generated from the rare-earth orbital magnetism in the perpendicularly magnetized iron garnets (Caretta et al., 2020), where the conventional heavy metal layers with strong spin-orbit coupling are not required.

In this work, we demonstrate two-dimensional (2D) reprogrammable spin wave networks by micromagnetic modeling. The proposed networks allow the unprecedented control of chiral exchange spin waves in chiral domain walls, overcoming two major challenges for realizing scalable magnonic circuits: curved and chiral transmission of spin waves. In such 2D spin wave networks, each cell can independently excite, transmit, and detect exchange spin waves with high speeds. The spin wave networks are scalable and can be two-dimensionally extended. The reprogrammabilities can be realized by manipulating the localized magnetic field. Furthermore, logic gates based on exchange spin waves can also be realized in such networks and both the prototypic XNOR gates as well as majority gates are demonstrated.

## Results and Discussion

### Chiral Exchange Spin Waves in Chiral Domain Walls

Reciprocal spin waves propagating in a Néel-type domain wall has been demonstrated in a non-DMI magnetic system (Körber et al., 2017). However, when the DMI is introduced, it will result in an asymmetric spin wave dispersion, which will lead to a non-reciprocal spin wave propagation as (Garcia-Sanchez et al., 2015):

(Equation 1) $$\omega =\sqrt{\omega_{k}\left(\omega_{k}-\omega_{⊥}+\frac{\pi \gamma D}{2\lambda M_{\text{S}}}\right)}\pm \frac{\pi \gamma Dk}{2M_{\text{S}}}\text{,}$$

where $\omega_{k}=2\gamma Ak^{2}/M_{\text{S}}$ is the exchange term and $\omega_{⊥}=2\gamma \mu_{0}dM_{\text{S}}/2\left(d+\pi \lambda \right)$ is the transverse anisotropy term, representing the dipolar interaction at the domain center. γ is the gyromagnetic ratio, *A* is the exchange constant, *k* is the wavevector, $M_{\text{S}}$ is the saturation magnetization, λ is the domain wall width, *d* is the film thickness, and *D* is the DMI parameter. In the exchange spin wave regime where the $k^{2}$ term dominates, the spin wave dispersion is quasi-quadratic and the DMI term makes linear modifications.

By placing a pair of magnetic nanowires on top of a magnetic insulator thin film with PMA and i-DMI, chiral exchange spin waves can be excited and propagate in the chiral domain walls. Owing to the dynamic dipolar coupling and the boundary condition formed by the double nanowires, only the spin waves with wave numbers k=nπ/a with *n* = 2, 4, 6… can be excited, where *a* is the distance between two identical nanowires. In spite of the high-order perpendicular standing spin waves, the interaction Hamiltonian can be described in the equation:

(Equation 2) $$\hat{H}/\hbar =\sum_{n}\left(g_{n}^{+k}\beta_{+k}\alpha^{†}+g_{n}^{-k}\beta_{-k}\alpha^{†}\right)\text{,}$$

where $\alpha^{†}$ is the magnon creation operator for the magnetic nanowires and $\beta_{+k}$ $\beta_{-k}$ are the magnon annihilation operators for spin wave modes along +*k* and -*k* direction in the domain wall. The interlayer dipolar interaction between magnetizations in magnetic nanowires and the domain wall can be calculated by the equation:

(Equation 3) $$g_{n}^{\pm k}=-\gamma \sigma_{n}\sqrt{\left(\mu_{0}M_{\text{S}}^{\text{N}}\right)\left(\mu_{0}M_{\text{S}}^{\text{F}}\right)}\int m_{\text{N}}^{\text{∗}}{\text{Λ}}_{\pm k}m_{\text{F}}e^{kx}\text{d}x\text{,}$$

where $\sigma_{n}=\frac{2}{n\pi }\sin\left(\frac{kw}{2}\right)\left(1-{\text{e}}^{-kh}\right)$, ${\text{Λ}}_{+k}=\left(\begin{array}{cc} -1 & -i\\ -i & 1 \end{array}\right)$, and ${\text{Λ}}_{-k}=\left(\begin{array}{cc} 1 & i\\ i & -1 \end{array}\right)$. The dynamic dipolar coupling $g_{n}^{+k}$ and $g_{n}^{-k}$ correspond to the spin wave propagating along +*k* and -*k* direction, respectively. $M_{\text{S}}^{\text{N}}$ and $M_{\text{S}}^{\text{F}}$ are the saturation magnetizations of the magnetic nanowire and the magnetic thin film. $m_{\text{N}}=\left({\left(\frac{a}{4hw}\sqrt{\frac{H_{0}+M_{\text{S}}^{\text{N}}N_{yy}}{H_{0}+M_{\text{S}}^{\text{N}}N_{xx}}}\right)}^{\frac{1}{2}},{\left(\frac{a}{4hw}\sqrt{\frac{H_{0}+M_{\text{S}}^{\text{N}}N_{xx}}{H_{0}+M_{\text{S}}^{\text{N}}N_{yy}}}\right)}^{\frac{1}{2}}\right)$ is the magnetization procession of the magnetic nanowire, whereas $N_{xx}$ and $N_{yy}$ are the demagnetization factors of the nanowire. $m_{\text{F}}=\left(m_{x}^{k},m_{y}^{k}\right)$ is the magnetization procession of spin wave modes in the chiral domain wall. In the exchange regime where $k^{2}$ dominates, the spin precession in the film is circular with $im_{x}^{k}=m_{y}^{k}=i{\left(\frac{1}{4d}\right)}^{\frac{1}{2}}$. The chiral spin wave propagation can be observed along the +*k* direction and -*k* direction due to $\left|g_{n}^{+k}\right|\neq \left|g_{n}^{-k}\right|$. When the magnetization of nanowires is antiparallel to the magnetization of the domain wall, the interlayer dipolar coupling reaches its maximum. Note that the chiral spin waves cannot be directly excited in the domain wall by non-magnetic antenna without the magnetic nanowires. For more details about the chiral excitation of exchange spin waves refer to Chen et al., 2018, Chen et al., 2019b, Yu et al., 2019.

We then use the micromagnetic solver OOMMF to demonstrate the chiral spin wave propagation in a chiral domain wall (Donahue & Porter, 1999). Unidirectional spin waves have been theoretically demonstrated in the Bloch-type domain walls, whereas extreme circumstances need to be fulfilled for realizing the chirality (Henry et al., 2019). Here, we use a pair of identical permalloy nanowries on top of a magnetic insulator thin film for exciting chiral spin waves in the chiral domain wall. The permalloy shows a saturation magnetization of 800 kA/m, an exchange constant 16 × 10^−12^ J/m, and a damping of 0.01 (Demidov et al., 2017). Two identical permalloy nanowires with the width of 20 nm, thickness of 4 nm, and a center-to-center distance of 40 nm are placed on top of the magnetic thin film. We select the TmIG as the magnetic thin film layer, which possesses the recently discovered i-DMI originated from the interface between the magnetic thin film and the garnet substrate (Avci et al., 2019, Ding et al., 2019). The saturation magnetization, exchange constant, and damping are set as 50 kA/m, 0.8 × 10^−12^ J/m, and 1 × 10^−4^, respectively. The i-DMI parameter is set as 0.05 mJ/m^2^, extracted from previous results (Shao et al., 2019). The i-DMI can determine the handedness of the Néel-type domain wall, as shown in the Figure S1. The anisotropy energy is set as 5 kJ/m^3^ where a Néel-type domain wall can be formed. The dimensions of the TmIG film are 60 μm × 200 nm × 4 nm (*xyz*). The damping of both ends of the film is set to 1 in order to avoid the reflected spin waves. Two permalloy nanowires are placed on the top center of the film. The cell sizes of the total simulation structure are 5 nm × 5 nm × 4 nm (*xyz*). No external magnetic field is applied in the simulation. The interlayer exchange coupling is neglected owing to the suppression of the spin wave chirality (Yu et al., 2019). A nanometer-thick insulating interlayer such as Al_2_O_3_ could be adapted in the real device for restraining the exchange interaction.

**Figure 1:**
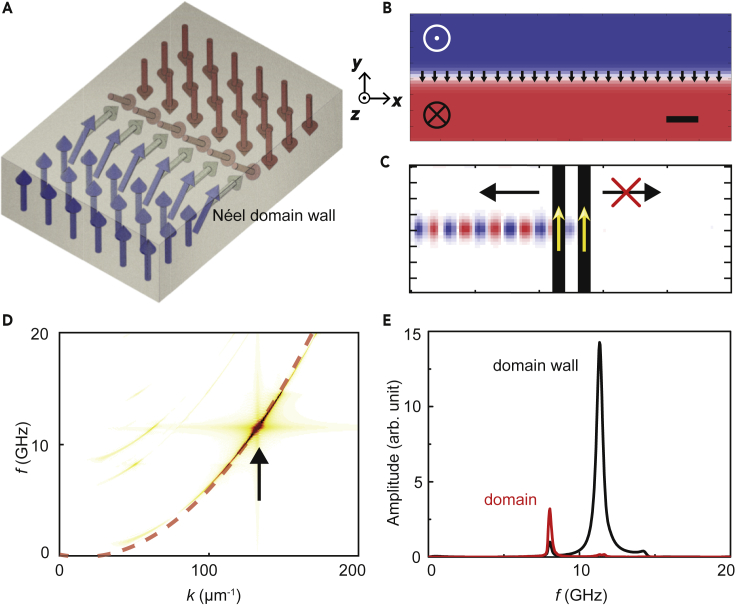
Chiral Spin Waves Propagation in Chiral Domain Wall (A) Schematic of the up-down (blue-red) domain structure with a Néel-type domain wall in between. (B) Domain structure of TmIG thin film extracted from the simulation. The scale bar is 50 nm. (C) The spatial map of the magnetization component $M_{z}$ at 5 ns with the spin wave resonance frequency of 11.33 GHz. The region of the permalloy nanowires is shown by black stripes, and the yellow arrows indicate the magnetic orientation in the nanowire. (D) Simulated spin wave dispersion relation by 2D FFT. The black arrow shows the highest excitation region, and the red dashed line is the calculated spin wave dispersion based on Equation 1. (E) 1D FFT of the spin wave excitation intensities of both domain wall and domain center.

First, we determine the equilibrium state of the hybrid simulation structure, where a Néel-type domain wall separates up-down domains in the TmIG thin film, as shown in Figure 1A. The middle part of the simulated structure with 0.5 μm length in *x* direction is shown in Figure 1B. This allows us to observe the ground state of the domain structure where the magnetizations in *z* direction are color coded. The magnetizations of the nanowires are antiparallel to those in the domain wall, for reaching the highest spin wave chirality. Subsequently, a uniform oscillating magnetic field is applied on the permalloy nanowires in the *x* direction according to Zhang et al. (2018):

(Equation 4) $$H_{\text{ex}}=H_{0}\frac{\text{sin}\left(2\pi f\left(t-t_{0}\right)\right)}{2\pi f\left(\text{t}-{\text{t}}_{0}\right)}\text{,}$$

where *H*_0_ = 0.2 mT and *f* = 20 GHz. The total simulation time is 5 ns with equidistant time steps of 25 ps. Furthermore, a 2D fast Fourier transformation (FFT) is conducted by using the magnetization components in the *x* direction. The obtained spin wave dispersion relation in the domain wall is shown in Figure 1D, along with the calculated dispersion based on Equation 1. The spin waves with mode number of *n* = 2 are excited and marked by a black arrow, and we observe the strongest excitation at 11.33 GHz with a wavenumber of *k* = 132.8 μm^−1^. The corresponding spin wave wavelength is around 47 nm, located in the exchange spin wave region (*λ* < 100 nm). The finite width of the nanowires and approximations in the analytic theory may lead to the discrepancy of spin wave wavelength between the simulation and the theory. The 1D FFT is also performed in both domain wall and domain center, shown in Figure 1E. The spin wave excitation efficiency in the domain wall is two orders of magnitude larger than that in the domain center at 11.33 GHz. More simulations of the weak spin wave propagation in the domain are shown in the Supplemental Information.

Therefore, a sinusoidal oscillating field is applied with the frequency of 11.33 GHz on the nanowires to excite exchange spin waves. The amplitude of the oscillating field is 0.2 mT. The group velocity of such exchange spin wave can be calculated by the derivative of the spin wave dispersion and $v_{g}$ = 1.254 km/s at 11.33 GHz. The spin wave decay length can be calculated by $l_{d}=\frac{v_{\text{g}}}{2\pi \alpha f}=1.76\times 10^{-4}$ m. The chiral coupling strengths of the spin wave modes can be calculated as $g_{2}^{+k}$ ≈ 0 and $g_{2}^{-k}$ ≈ 3.7 GHz, indicating a chiral spin wave propagation in -*x* direction. The frequency non-reciprocity introduced by the i-DMI term in the dispersion relation does not contribute to the chiral emission of exchange spin waves. The spatial map of the magnetization components in *z* direction $M_{z}$ at 5 ns is shown in Figure 1C. An obvious chirality of spin wave propagation in the chiral domain wall can be observed. Note that we only discuss the chiral spin wave with the frequency of 11.33 GHz in this work, as a demonstration of the reprogrammable spin wave networks. Moreover, directions of the spin wave propagating in the chiral domain wall can be completely reversed when the domain wall magnetization is reversed (see Supplemental Information for more details). In the forthcoming chapter, we discuss the 2D spin wave networks based on chiral exchange spin waves and chiral domain walls.

### Spin Wave Propagation in Networks

**Figure 2:**
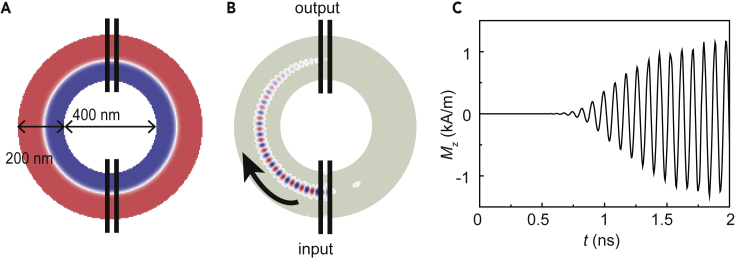
Spin Waves Propagation in a Ring-Shape Structure (A) Sketch of a ring-shaped waveguide with a Néel-type domain wall in the center of the up-down domains. Two pairs of permalloy nanowires are placed on the ring-shaped waveguide as input and output. The inner and outer diameters of the waveguide are 400 and 800 nm, respectively. (B) A snapshot of the magnetization component $M_{z}$ at 0.75 ns. (C) The time-dependent magnetization component $M_{z}$ in the domain wall center underneath the output nanowires.

Next, we demonstrate the exchange spin wave propagation in a TmIG ring-shaped waveguide, shown in Figure 2A. The up-down domains are set manually in the ring-shaped waveguide where a Néel-type domain wall separates the neighboring domains. The realization of the spin wave turning a corner is the major challenge for building complex spin wave networks. Previous efforts applied a continuous Oersted field generated by an electrical current to guide a curved trajectory for spin waves (Vogt et al., 2014). In contrast, energy losses and Joule heating are generated owing to the current, which neutralize the advantage of low-power-consumption property provided by spin waves. However, the spin wave propagation in Néel-type domain walls in curved structures can be transmitted efficiently (Garcia-Sanchez et al., 2015). In the proposed ring-shaped structure, the inner and outer diameter are 400 and 800 nm, respectively. A pair of identical permalloy nanowires with the width of 20 nm and the center-to-center distance of 40 nm is set as the input. Same permalloy nanowires are placed on the other end of the ring waveguide as the output. The domain structures can be written and rewritten by localized external magnetic fields, indicating the reprogrammability of the chiral domain wall-based spin wave networks (see Supplemental Information for the reprogrammability of the domain structure using external field). Recently discovered thermally assisted scanning probe lithography can also be used to pattern the spin textures (Albisetti et al., 2016), which might replace the external field for the reprogrammability. Chiral exchange spin waves are excited by a sinusoidal oscillating field with the frequency of 11.33 GHz on the input nanowires. The oscillating magnetic field is only localized at the input nanowires, and the angle between the nanowire and the curved domain wall in Figure 2A is around 88°, similar to the straight domain wall (exactly 90°). In the simulation we find that the amplitude and the unidirectionality of the exchange spin waves are almost consistent in both conditions. A snapshot of the magnetization component $M_{z}$ at 0.75 ns is shown in Figure 2B. Chiral exchange spin waves can be observed propagating inside the domain wall from the input to the output. The time-dependent magnetization component $M_{z}$ in the domain wall underneath the output nanowires is shown in Figure 2C. One can observe that it takes around 0.75 ns for spin waves to transmit from the input to the output, and the spin wave signal strength stabilizes at around 1.75 ns.

**Figure 3:**
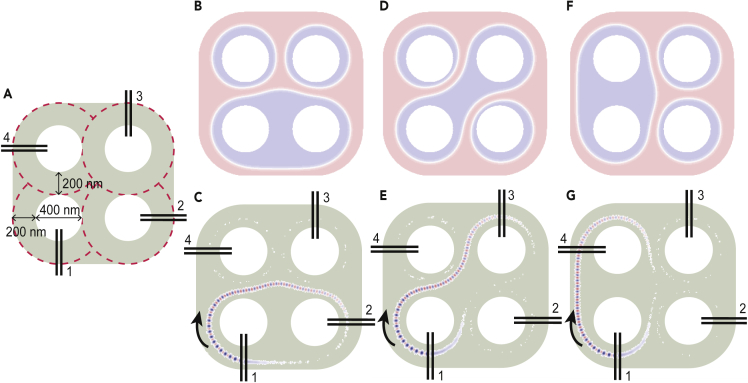
2D Spin Wave Networks in an Anti-Dot System (A) Sketch of the 2 × 2 network with four ring-shaped waveguides marked from cell 1 to cell 4. Four pairs of permalloy nanowires are placed on the TmIG waveguide. Dimensions of the waveguide are marked. (B) Domain structure where domain walls connect cell 1 with cell 2. (C) Communication of cell1 and cell 2 via exchange spin waves in chiral domain walls. (D) Domain structure where domain walls connect cell 1 with cell 3. (E) Communication of cell1 and cell 3 via exchange spin waves in chiral domain walls. (F) Domain structure where domain walls connect cell 1 with cell 4. (G) Communication of cell1 and cell 4 via exchange spin waves in chiral domain walls.

After the demonstration of the chiral spin wave propagation in the domain wall of a ring-shaped structure, we now investigate the chiral domain walls-based 2D spin wave networks. Considering a ring-shaped structure as one unit cell, the network can be two-dimensionally extended. An example of the 2 × 2 network is shown in Figure 3A where four identical rings are placed in a quadrangle condition. The anti-dot system is chosen where domain walls are not expelled from the track owing to the shape anisotropy caused by confined boundaries. The dimensions of the structure are marked in the figure. Four groups of identical permalloy nanowires are placed on different rings, labeled as 1, 2, 3, and 4. Different domain structures can be stabilized by localized external fields, whereas domain walls can connect cell 1 with cell 2 (Figure 3B), cell 3 (Figure 3D), or cell 4 (Figure 3F). In all these three conditions, chiral exchange spin waves with the frequency of 11.33 GHz are excited in cell 1. In the 2D network, cells can communicate with each other by sending and receiving spin waves where the information carried by spin waves can be exchanged in the connected cells. Snapshots of the time-dependent magnetization component $M_{z}$ at 1.8 ns are shown in Figures 3C, 3E, and 3G, and it allows us to probe that spin waves only propagate in the domain wall. Note that a weak mode without the phase oscillations exists in the counter direction of the unidirectional spin waves, which could originate from the fluctuation of dynamic dipolar fields generated by the magnetic nanowires.

### Spin Wave Logics Based on Networks

**Figure 4:**
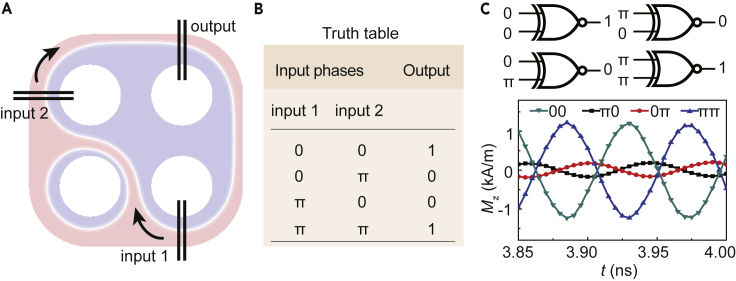
Spin-Wave-Based XNOR Logic Gate (A) Domain structure of the 2 × 2 network with two inputs and one output. (B) The truth table of the XNOR logic gate based on the exchange spin waves in the chiral domain wall. (C) Time-dependent magnetization component $M_{z}$ in the domain wall center underneath the output nanowires with four input logics.

Logic computing can also be realized in the 2D spin wave networks, which would benefit for building magnonic logic devices. Figure 4A shows the domain structure where the up domain expands to three cells and the domain wall connects these cells. Three groups of permalloy nanowires serve as two inputs and one output, placed on those three connected cells. Chiral exchange spin waves are excited in two inputs and detected in the output. Two phase shifters and two attenuators are mounted on two inputs separately. When the exchange spin waves with the frequency of 11.33 GHz travel through the domain wall below two permalloy nanowires, the spin wave intensity will lose a factor of around $\frac{1}{3}$ where the energy is transferred to the nanowires via dynamic dipolar coupling (see Supplemental Information for more simulations about the spin wave attenuation). Owing to the certain spin wave travel distance, an intrinsic phase difference between input 1 and input 2 exists that results in 0.23π in the simulation. This means that a spin wave with a phase shift of 0.23π from input 1 and a spin wave with a phase shift of 0 from input 2 are in-phase. The intrinsic phase of spin waves can be simply controlled by setting the initial time delay, and the delay time of the phase shift 0.23π can be set as 10.15 ps. In reality, phase shifters can be implemented for controlling the initial phase of the injected microwave (Talmelli et al., 2019). The amplitude of the excitation oscillating field is set to 0.3 mT, and the intrinsic phase is set to 0.23π for input 1. The amplitude of the excitation oscillating field is set to 0.2 mT for input 2. By choosing different amplitudes of the oscillating fields, two branches of spin waves excited by two inputs have a very close amplitude when they arrive at the output. The starting phases of the input 1 and input 2 are defined as 0, as shown in the truth table in Figure 4B. The constructive interference can be formed in this condition and the logic output becomes 1. Sinusoidal oscillating fields with the frequency of 11.33 GHz are applied in both inputs simultaneously. After the spin waves from input 1 arrive at the output with a uniform strength (around 3.5 ns), a stable logic output can be realized. When a phase shift of π is applied in input 1 or 2, the destructive interference is formed and the logic output is 0. Figure 4B shows the truth table of the spin wave-based XNOR logic gate. The spin wave signals in the four proposed output states (extracted in the domain wall center) are shown in Figure 4C, revealing the XNOR logic gate functionality. The ON/OFF ratio of the XNOR gate is 7.02. More accurate selection of the amplitudes of oscillating fields as well as the initial phases in two inputs could further enhance the ON/OFF ratio.

**Figure 5:**
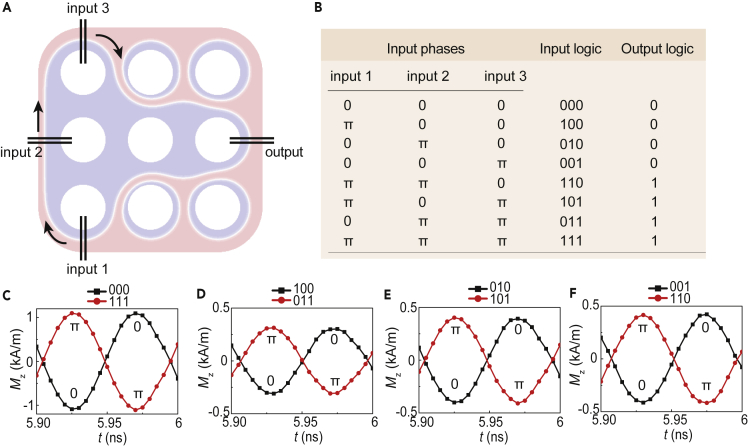
Spin-Wave-Based Majority Gate (A) Domain structure of the 3 × 3 network with three inputs and one output. (B) The truth table of the majority logic gate based on the exchange spin waves in the chiral domain wall. (C) Time-dependent magnetization component Mz in the domain wall center underneath the output nanowires with inputs of "000" and "111". (D) Magnetization component Mz with inputs of "100" and "011". (E) Magnetization component Mz with inputs of "010" and "101". (F) Magnetization component Mz with inputs of "001" and "110".

Owing to the scalability of the spin wave network, more complex logic functionalities can be realized. A spin wave majority gate is proposed in a 3 × 3 network, where the domain structure is shown in Figure 5A. The majority logic is widely employed in CMOS-based logic circuits and could improve the efficiency of circuits by reducing circuit complexities (Klingler et al., 2014, Kanazawa et al., 2017). Three inputs are placed in three neighboring cells, and the output is placed in the other end of the network. The chiral domain wall connects the three inputs and the output together where spin waves can transport. Previously, it is demonstrated that the spin wave intensity will lose a factor of $\frac{1}{3}$ when traveling through the area below two permalloy nanowires. The intrinsic spin wave phase difference between input 1 and input 2 (input 2 and input 3) is 0.63π. We manually eliminate the phase differences by switching the initial phases, so the spin waves from three inputs are excited in-phase, as the input logic “000.” The input phases can be controlled by phase shifters mounted on three inputs. Excitation amplitude of the sinusoidal oscillating fields for input 1, input 2, and input 3 are set to 0.18, 0.12, and 0.08 mT, respectively, to equalize the intensities of the output signals. A table of the majority gate logic based on the input phases is shown in Figure 5B. In the spin wave majority gate, a logic output “0” is defined as a certain phase φ and a logic output “1” is defined as φ+π. Output signals observed with different logics from 5.9 to 6 ns are shown in Figures 5C–5F. Therefore, a phase-based spin wave majority gate can be realized with the output phase representing the majority of input phases. More complex spin wave logics can be designed and realized by the two-dimensional extension of such networks.

### Conclusion

In summary, we have demonstrated scalable networks based on chiral exchange spin waves and chiral domain walls. The proposed nanoscale spin wave networks are reprogrammable via the external magnetic field. Logic gate functions can be realized in such spin wave networks. By using the equipment such as the oscilloscope and the time domain reflectometry, the simulated effects can be electrically detected and the logic circuit can cascade different logic gates. In the proposed devices, exchange spin waves are efficiently channeled in the domain wall center, acting as a local potential well for spin waves. Compared with the spin waves traveling in the plane film, the localized modes have more immunity to defects. Stabilizing the environment temperature and the reduction of input powers could contribute to the robustness of the thermal fluctuation. Such high-speed, compact, and low-power magnonic networks are proved to show high performance, and our findings may raise prospects for exploring the extensive applications of spin wave-based nano-circuits.

### Limitations of the Study

Current nano-fabrication technologies are challenging to realize the proposed magnonic networks in the sub-50-nm scale. Additionally, the localized external field is required to precisely create the arbitrary magnetic patterns in the anti-dot system.

### Resource Availability

#### Lead Contact

Further information and requests for resources should be directed to and will be fulfilled by the Lead Contact, Jilei Chen (remichen@buaa.edu.cn).

#### Materials Availability

This study did not generate new materials.

#### Data and Code Availability

The datasets generated during this study are available at Mendeley Data: https://doi.org/10.17632/6wr9h77z54.2.

## Methods

All methods can be found in the accompanying Transparent Methods supplemental file.

## Supplemental Information

Document S1. Transparent Methods and Figures S1–S6

## Footnotes

Supplemental Information can be found online at https://doi.org/10.1016/j.isci.2020.101153.
