# Supplementary material for: Chiral Magnonics: Reprogrammable Nanoscale Spin Wave Networks Based on Chiral Domain Walls
Source: iScience. 2020 May 12;23(6):101153. doi: 10.1016/j.isci.2020.101153 (PMC7251948; doi:10.1016/j.isci.2020.101153)
Supplement: Document S1. Transparent Methods and Figures S1–S6 [file mmc1.pdf]

iScience, Volume *23*

Supplemental Information

Chiral Magnonics: Reprogrammable Nanoscale Spin Wave Networks Based on Chiral Domain Walls

Jilei Chen, Junfeng Hu, and Haiming Yu

I. Simulations of different i-DMI parameters in the TmIG film

II. Spin wave propagation in the domain

III. Reversed unidirectional spin wave propagation in the chiral domain wall

IV. Reprogrammability of the domain structure by external magnetic fields

V. Spin wave attenuation through the region underneath two permalloy nanowires

VI. Spatial maps of the spin wave logics based on a 2×2 network

Transparent Methods

Micromagneitic simulations

The micromagnetic simulations are performed by OOMMF (http://math.nist.gov/oommf) with finite difference method. The time-dependent magnetization is implemented a first order forward Euler method with a step size control on the Landau-Lifshitz-Gilbert equation:

$$\frac{d\mathbf{M}}{dt} = -|\gamma|\mathbf{M} \times \mathbf{H}_{\text{eff}} + \frac{\alpha}{M_{\text{S}}}(\mathbf{M} \times \frac{d\mathbf{M}}{dt}), \tag{S1}$$

where $\mathbf{M}$ is the magnetization, $\mathbf{H}_{\text{eff}}$ is the effective magnetic field, γ is the gyomangnetic ratio and α is the Gilbert damping. To stimulate the magnetization dynamics in the permalloy nanowires, a uniform excitation field in the x direction is applied following the equation:

$$H_{\text{ex}} = H_0 \frac{\sin(2\pi f(t - t_0))}{2\pi f(t - t_0)}, \tag{S2}$$

where H_0 is set as 0.2 mT, f=20 GHz and t_0 =100.1 ps. The sine cardinal (sinc) function promises the iso-strength excitation from -20 GHz to 20 GHz. The simulation period is 5 ns for one fixed field, with an equivalent step of 25 ps.

1D and 2D fast Fourier transformations

After extracting the time-dependent the magnetization component in the x directions, 1D FFT is performed for treating data from time domain to the frequency domain, allowing to obtain the amplitude and frequency of the spin wave modes. Meanwhile, the 2D FFT in both time and the x direction is performed. The information of the wavevector k can be extracted and the spin wave dispersion relation can be determined.

I. Simulations of different i-DMI parameters in the TmIG film

The up-down domain structures are simulated with different i-DMI parameters with D=0.05 mJ/m^2 in fig. S1(a), D=0 mJ/m^2 in fig. S1(b) and D=-0.05 mJ/m^2 in fig. S1(c). The thickness of the simulated structure is 4 nm with the cell sizes of 1 nm $\times$ 1 nm $\times$ 1 nm. Each cone in the figure corresponds to the spin in one cell. One can observe that the i-DMI contributes to form the Néel type domain wall while the Bloch type domain wall is formed without i-DMI. When the i-DMI parameter changes the sign, the orientations of spins in the domain wall will reverse.

II. Spin wave propagation in the domain

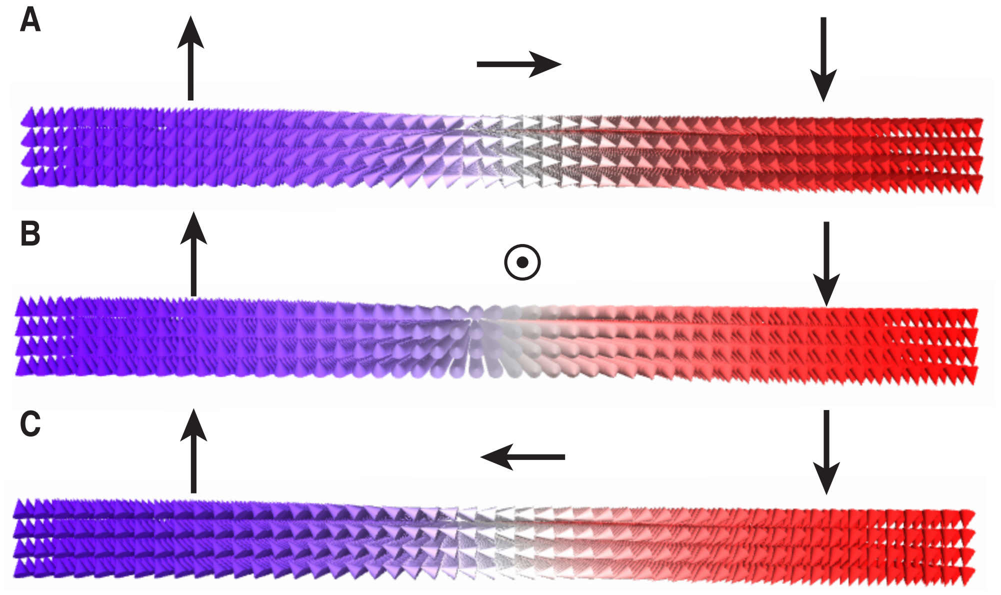

FIG. S1. Up-down (blue-red) domain structures with (A) D=0.05 mJ/m^2 (B) D=0 mJ/m^2 and (C) D=-0.05 mJ/m^2. Each cone corresponds to the spin in the 1 nm $\times$ 1 nm $\times$ 1 nm TmIG cell. Related to Figure 1.

Fig. S2 shows the modelling of the spin wave propagation in the domain. The simulated structure is same as the one shown in fig. 1 in the main text, while the domain center is analyzed rather than the domain wall. The spin wave dispersion relation obtained by the 2D FFT is shown in fig. S2 (c) while a frequency gap of the bulk mode is shown. The main resonance frequency is at 8.05 GHz. When a oscillating field with the frequency of 8.05 GHz is injected into the permalloy nanowires, the spin waves will propagate in both direction in the domain, shown in the spatial map in S2 (b).

III. Reversed unidirectional spin wave propagation in the chiral domain wall

Fig. S3 shows the modelling of the spin wave propagation in the reversed direction in the chiral domain wall. The equilibrium state of the hybrid simulation structure is the down-up domain state with a Néel type domain wall in between. We could observe the ground state of the domain structure in Fig. S3(a) where the magnetizations in z direction are color-coded. The spins inside the domain wall point from the up domain to the down domain due to the positive i-DMI parameter. The magnetizations in the domain wall and the permalloy nanowires are in the parallel configuration. The sinc pulse induced spin waves with mode number of n=2 are excited which is marked by a black arrow in Fig. S3(c), and one could

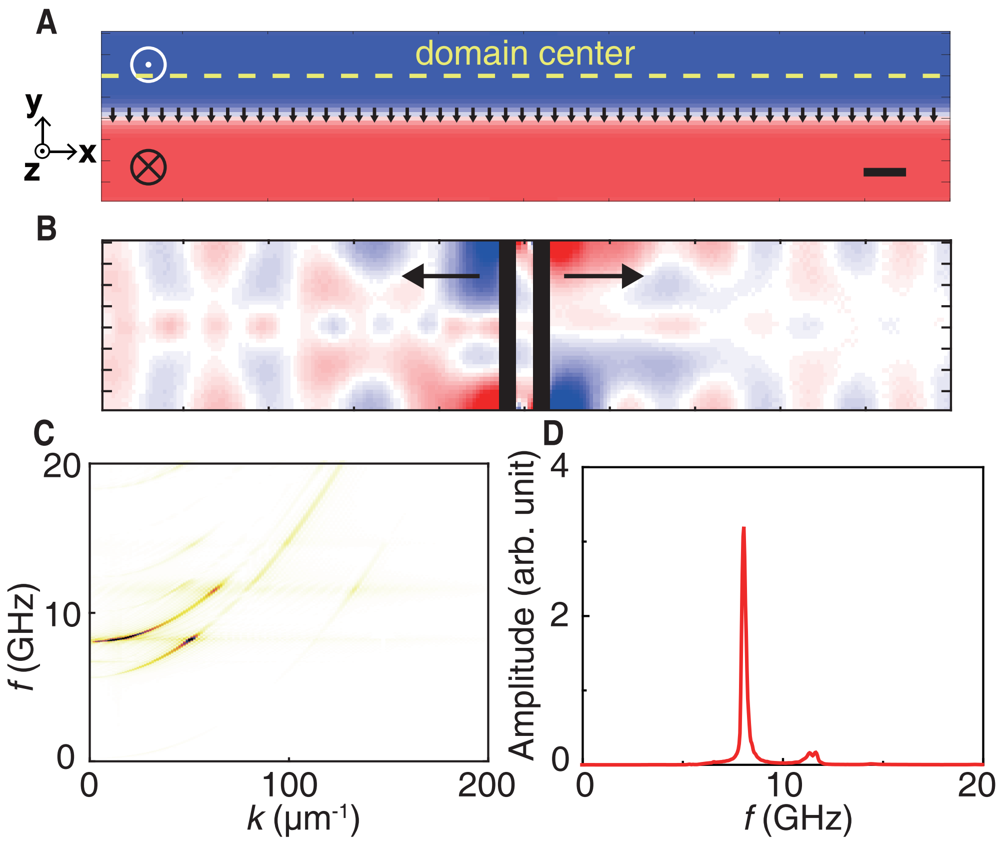

FIG. S2. (A) Schematic of the up-down (blue-red) domain structure with a Néel type domain wall in between. The scall bar is 50 nm long. (B) The spatial map of the magnetization component variations in x direction at 4 ns, with the excitation frequency of 8.05 GHz. (C) Simulated spin wave dispersion relation by 2D FFT in the domain center at the position marked in (A). (D) 1D FFT of the spin wave excitation intensities the domain center. Related to Figure 1.

observe the strongest excitation at 11.25 GHz. The frequency shift of the strongest response between the parallel and antiparallel configurations comes from the i-DMI induced frequency non-reciprocity in the dispersion relation. 1D FFT is also performed in the domain wall, shown in Fig. S3(d). Then an sinusoidal oscillating field is applied with the frequency of 11.25 GHz on the nanowires to excite exchange spin waves and the spatial map of the magnetization components in z direction M_z at 5 ns is shown in Fig. S3(b). An obvious reversed unidirectionality of spin wave propagation from the main text is shown, indicating the programmable spin wave propagating directions.

IV. Reprogrammability of the domain structure by external magnetic fields

The domain structures can be written and rewritten by localized external magnetic fields

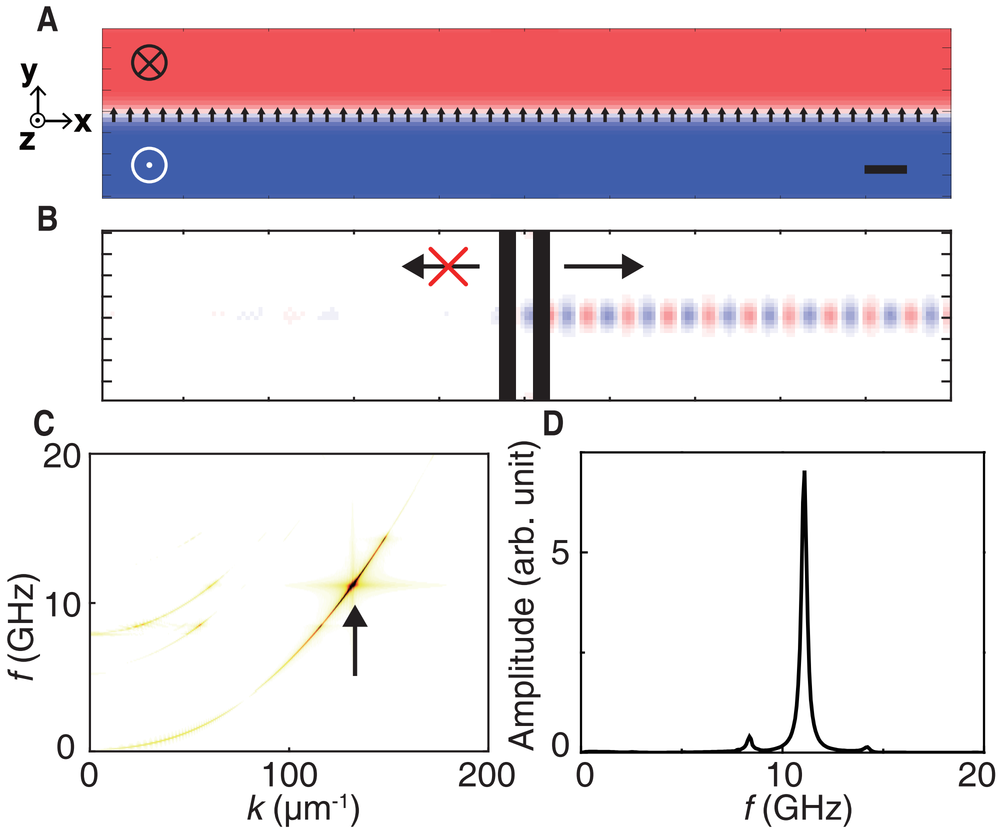

FIG. S3. (A) Schematic of the down-up (red-bule) domain structure with a Néel type domain wall in between. The spins inside the domain wall point from the up domain to the down domain due to the positive i-DMI parameter. The scall bar is 50 nm long. (B) The spatial map of the magnetization component variations in z direction at 5 ns, with the excitation frequency of 11.25 GHz. (C) Simulated spin wave dispersion relation by 2D FFT in the domain wall. (D) 1D FFT of the spin wave excitation intensities the domain wall. Related to Figure 1.

in the ring-shape waveguide, shown in Fig. S4. The initial state of the domain structure is shown in Fig. S4(a) with a Néel type domain wall seperating the up domain and down domain. Then a global external field of -250 mT in z direction is applied to fully saturate the system. After removing the external field, the ground state is shown in Fig. S4(b). A well defined external field of 200 mT in z direction with the radius of 250 nm in the center of ring-shape structure is applied, forming the centering up domain in Fig. S4(c). Finally the external field is removed and the equilibrum state is shown in Fig. S4(d).

V. Spin wave attenuation through the region underneath two permalloy nanowires

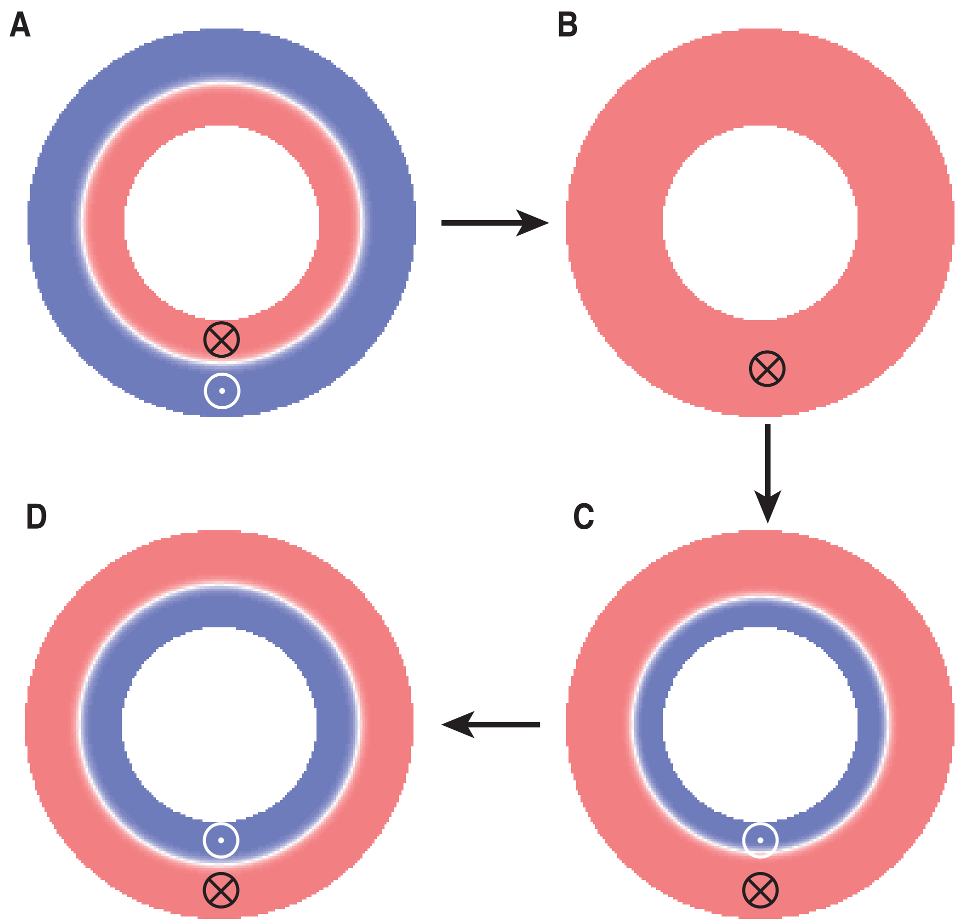

FIG. S4. The reprogrammable procedures of the ring-shape waveguide with four states. Initial state (A), the state with a global external field of -250 mT (B), the state with well defined field of 200 mT (C) and the final state (D) are shown. Related to Figure 2.

Fig. S5 shows the spin wave signal in the chiral domain wall underneath a pair of permalloy nanowires. The exchange spin waves with the resonance of 11.33 GHz is transmitted from positive to the negative positions in the horizontal direction. A pair of permalloy nanowires is place around 0 nm shown in the figure. When the spin waves pass through, the energy will be transferred in to the nanowires due to the dipolar coupling, causing the intensity attenuation of the spin waves. The attenuation factor is around 1/3 obtained from the spin wave signal.

VI. Spatial maps of the spin wave logics based on a 2×2 network

Fig. S6 shows spatial maps of the spin wave logics with the input phases of $\pi 0$ Fig. S6 (a) and $\pi\pi$ Fig. S6 (b). The destructive and constructive interferences can be obtained by

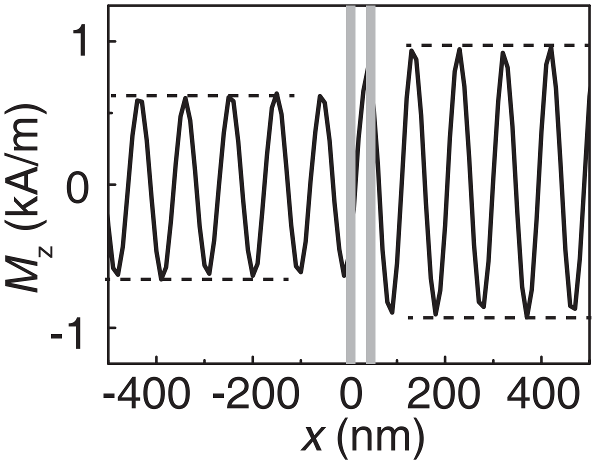

FIG. S5. Spin wave signal in the straight chiral domain wall, propagating from positive to negative in the x direction. The region of two identical permalloy nanowires are marked in the figure. Related to Figure 4.

the ouput signal, which demonstrates the XNOR logic gate functionality.

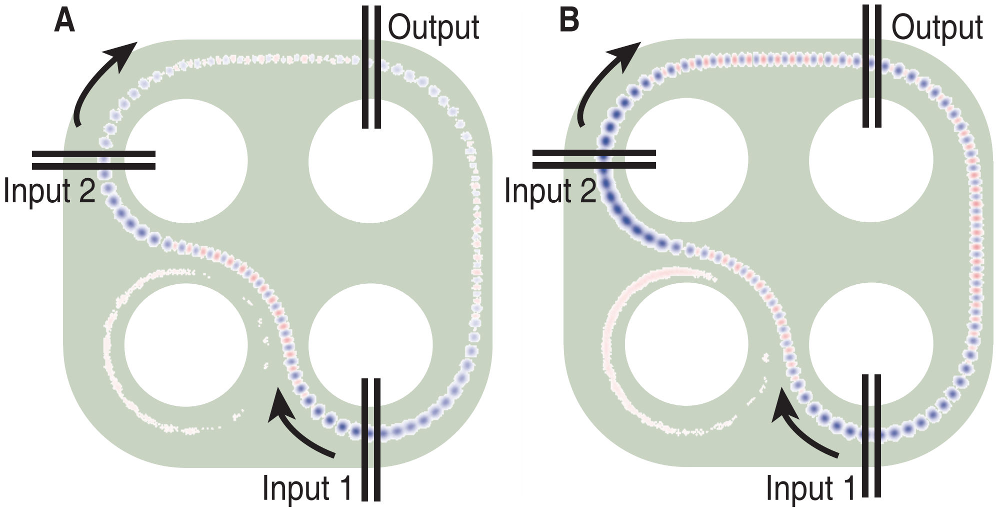

FIG. S6. Snapshots of the magnetization variation M_z at 4 ns with the input phases as $\pi 0$ (A) and $\pi\pi$ (B) based on a 2×2 network. The outputs are then obtained as 0 and 1, respectively. Related to Figure 4.
